# Optimization of Protoplast Isolation and Transformation for a Pilot Study of Genome Editing in Peanut by Targeting the Allergen Gene *Ara h 2*

**DOI:** 10.3390/ijms23020837

**Published:** 2022-01-13

**Authors:** Sudip Biswas, Nancy J. Wahl, Michael J. Thomson, John M. Cason, Bill F. McCutchen, Endang M. Septiningsih

**Affiliations:** 1 Department of Soil and Crop Sciences, Texas A&M University, College Station, TX 77843, USA; sudipbmb@tamu.edu (S.B.); nwahl@utk.edu (N.J.W.); m.thomson@tamu.edu (M.J.T.); 2 Texas A&M AgriLife Research and Extension Center at Stephenville, Stephenville, TX 76401, USA; john.cason@ag.tamu.edu (J.M.C.); bill.mccutchen@ag.tamu.edu (B.F.M.)

**Keywords:** peanut (*Arachis hypogaea* L.), gene editing, CRISPR–Cas9, *Ara h 2*, protoplast, transformation efficiency

## Abstract

The cultivated peanut (*Arachis hypogaea* L.) is a legume consumed worldwide in the form of oil, nuts, peanut butter, and candy. Improving peanut production and nutrition will require new technologies to enable novel trait development. Clustered regularly interspaced short palindromic repeats and CRISPR-associated protein 9 (CRISPR–Cas9) is a powerful and versatile genome-editing tool for introducing genetic changes for studying gene expression and improving crops, including peanuts. An efficient in vivo transient CRISPR–Cas9- editing system using protoplasts as a testbed could be a versatile platform to optimize this technology. In this study, multiplex CRISPR–Cas9 genome editing was performed in peanut protoplasts to disrupt a major allergen gene with the help of an endogenous tRNA-processing system. In this process, we successfully optimized protoplast isolation and transformation with green fluorescent protein (GFP) plasmid, designed two sgRNAs for an allergen gene, *Ara h 2*, and tested their efficiency by in vitro digestion with Cas9. Finally, through deep-sequencing analysis, several edits were identified in our target gene after PEG-mediated transformation in protoplasts with a Cas9 and sgRNA-containing vector. These findings demonstrated that a polyethylene glycol (PEG)-mediated protoplast transformation system can serve as a rapid and effective tool for transient expression assays and sgRNA validation in peanut.

## 1. Introduction

Cultivated peanut or groundnut (*Arachis hypogaea L.*) is an allotetraploid (2n = 4x = 40) with a large reservoir of seed oil (~46–58%) and high-quality protein (~22–32%) [1]. In 2018, about 45.95 million tons of peanut were produced across 28.51 million ha worldwide (FAO, Rome, Italy, 2018). China and India are the leading peanut producers globally, while the USA is fifth. Traditional peanut breeding has been a lengthy process with difficulties due to polyploidy and sterility barriers [2]. However, the availability of the recently published complete peanut genome [3,4] and bioinformatics resources, such as the peanut genome database [5], has enabled more rapid progress in peanut genetics, genomics, and molecular breeding [6,7,8,9,10,11,12,13,14]. Furthermore, the implementation of functional genomics combined with biotechnology, especially DNA recombinant technology, will serve as an essential tool to further enable the discovery and characterization of genes of agronomic importance and speed up the progress in peanut breeding efforts. Unlike *Arabidopsis* and rice, making transgenic peanut plants through *Agrobacterium* transformation is more challenging and has a lower efficiency [15]. In this case, *Agrobacterium rhizogenes* has been frequently used for the transformation of hairy roots in peanuts [16], but there is no report on generating mature plants from the transformed root. However, some products of transgenic research on peanuts have been developed via *Agrobacterium tumefaciens*-mediated transformation, including varieties having resistance to some biotic stresses, such as viruses [17], insects [18] and fungi [19], and tolerance to abiotic stresses, such as drought and salt salinity [20]. Finally, allergen-reduced peanut with improved grain quality has also been developed via RNAi by knocking out the *Ara h 2* gene [21].

Among the recent techniques in biotechnology, genome editing is the most promising technology to study gene functions and help speed up crop improvement. Gene editing is a versatile technology that can be used to more precisely knock out the function of a gene [22,23], inactivate undesirable chromosomal DNA [24], and regulate endogenous genes [25], among other applications. Thus far, three genome-editing techniques have been established: zinc-finger nucleases (ZFNs), transcription activator-like effector nucleases (TALENs), and clustered regularly interspaced short palindromic repeats associated with nuclease Cas9 (CRISPR–Cas9) [26]. Among them, CRISPR–Cas9 genome editing has proven to be the most popular and widely used for its precision, effectiveness, and ease; moreover, this technology can be applied in both diploid and polyploid plants [2].

Despite its economic importance, peanut is less amenable to genome-editing technology than other crops, such as rice, maize, and wheat; therefore, testing and evaluating this technology is an important step. As generating stable genome-edited plants is complex and labor intensive [27], it is necessary to evaluate the most effective Cas9-gRNA beforehand. To evaluate the potential of the CRISPR–Cas9 system in peanuts, a reproducible system for the design, construction, and delivery of Cas9-gRNA needs to be developed and validated via in vitro and in vivo systems. For an in vivo assay, protoplast transformation can be used as a tool to express genes transiently as well as evaluate the genome-editing efficacy [28,29].

To develop this system, we targeted a peanut allergen gene. Allergenicity to peanuts is one of the most life-threatening food allergies and one of the most challenging problems faced by peanut breeders and researchers. This problem negatively impacts the peanut and food industries, and its significant health consequences demonstrate the dire need to find a solution for this problem. A total of 12 proteins are potentially involved in peanut allergenicity, four of which have been identified as the most important based on clinical tests [30]. Here, we targeted a major allergen gene, *Ara h 2*, for optimizing gene editing in peanut protoplasts. Since the initial successful isolation of peanut protoplasts about four decades ago [31], there have been limited reports on the application of protoplasts in peanuts, primarily due to relatively low yields of the protoplasts. In this study, we describe a simple and efficient protocol for the isolation of peanut protoplasts and its application for transient gene expression studies and sgRNA validation for gene editing.

## 2. Results

### 2.1. An Efficient Method of Protoplast Isolation from Peanut Seedlings

**Figure 1:**
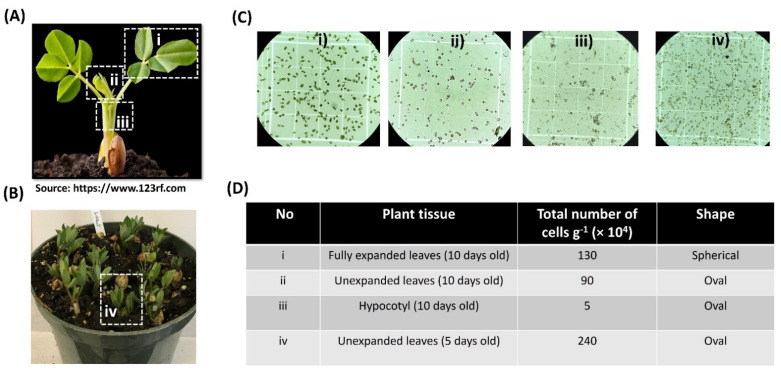
Protoplast isolation from different tissues and ages of peanut seedlings: (**A**) 10 days old; (**B**) 5 days old peanut seedlings; (**C**) protoplast from different tissues (i, ii, iii, and iv) of A and B; (**D**) the total number of protoplasts and their shapes from different tissues of peanut seedlings.

Protoplast transformation is a convenient and reliable system to optimize gene editing in plants [27]. It represents a key validation component of an efficient gene-editing pipeline (Figure S1). Selecting the proper source of plant tissue is the first critical step for obtaining a high yield of protoplasts. In this study, we isolated protoplasts from different tissues of peanut seedlings (Figure 1A,B). The yield of cells from fully expanded leaves (section i) of 10 days old peanut seedlings was higher than those of unexpanded leaves (section ii) and hypocotyl (section iii), but the shape of the protoplasts from section i was spherical (Figure 1C). From both sections ii and iii, we found oval-shaped protoplasts, although the protoplast yields were much lower, especially from section iii (Figure 1D). Moreover, we also compared the protoplast yields of the unexpanded leaves from 5 days old peanut seedlings (section iv; Figure 1B). The results showed that the yield of protoplasts isolated from 5 days old seedlings was higher than that of the 10 days old peanut seedlings (Figure 1C,D). Considering protoplast yield and shape, unexpanded leaves from 5 days old seedlings have been the most suitable source of plant tissue.

### 2.2. Temperature Effect on Protoplast Viability and Testing Constitutive Promoters

**Figure 2:**
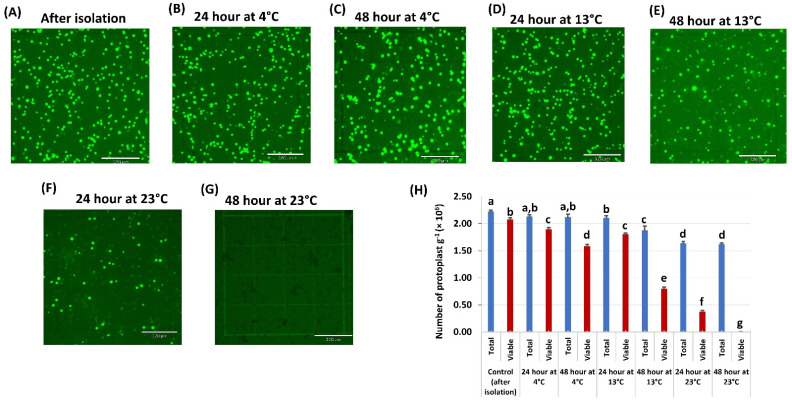
Protoplast viability assay under different temperatures: (**A**–**G**) micrograph of viable protoplasts stained with FDA under fluorescence field kept at three different temperatures, 4 °C, 13 °C, and 23 °C, for 24 h and 48 h; (**H**) the effects of temperature on protoplasts. The number of total protoplasts and viable protoplasts were counted after 24 h and 48 h cultivation. Values represent means ± SE (*n* = 7). The different letters indicate significant differences at *p* < 0.05.

Temperature plays a crucial role in protoplast viability. We kept and tested the protoplast viability at three different temperatures (4 °C, 13 °C, and 23 °C) after isolation. The results showed that the number of both total and viable protoplasts decreased as the temperature increased (Figure 2). There were more viable protoplasts at 4 °C than other temperatures. Unfortunately, all the protoplasts died at 23 °C for 48 h. The protoplast at 13 °C for 24 h showed a similar viability rate as 4 °C, although the viability was drastically decreased after 48 h. For further experiments, we selected the condition at 13 °C for 24 h as an ideal condition for peanut protoplast transformation because we found the highest transformation efficiency and viability with CmYLCV:GFP plasmid (data not shown). Although the protoplast showed the highest viability at 4 °C, we did not find any GFP expression even after 96 h of transformation.

We also tested the two constitutive promoters’ activity (35S and CmYCLV promoters) in peanut protoplasts and found that protoplasts transformed with CmYLCV:GFP gave a higher transformation efficiency than 35S:GFP based on the number of GFP expressed protoplasts (Supplementary Figure S2). Therefore, CmYLCV:GFP plasmid was used for further optimization.

### 2.3. Effects of PEG Concentration on Protoplast Transformation Efficiency and Viability

**Figure 3:**
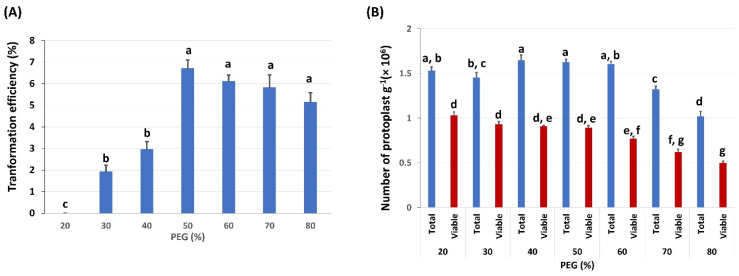
Effect of PEG concentration on protoplast transfection: (**A**) the transformation efficiency (TE) of protoplasts cultivated with various concentrations of PEG. TE was calculated after 24 h cultivation; (**B**) the effects of PEG concentration on the number of protoplasts. The number of total protoplasts and viable protoplasts counted after 24 h cultivation. Values represent means ± SE (*n* = 7). The different letters indicate significant differences at *p* < 0.05.

Polyethylene glycol (PEG) is widely used to directly deliver DNA or plasmids into individual plant cells or protoplasts. We tested the effects of different PEG 4000 concentrations on protoplast transformation efficiency, with concentrations (*w*/*v*) ranging from 20% to 80% (Figure 3; Supplementary Figure S3). In each treatment, the different PEG concentrations were tested with the optimal DNA and 5 min DNA incubation time. Additionally, the effect of PEG concentrations on protoplast viability was also tested. After 5 min PEG incubation and 24 h cultivation, it was evident that the 50% PEG concentration yielded the highest transformation efficiency (TE) up to 7% (Figure 3A). The numbers of total intact and viable protoplasts decreased as the PEG concentration increased (Figure 3B), which partly might be caused by the PEG-induced high permeability.

### 2.4. Effects of Plasmid Concentrations on Transformation Efficiency

**Figure 4:**
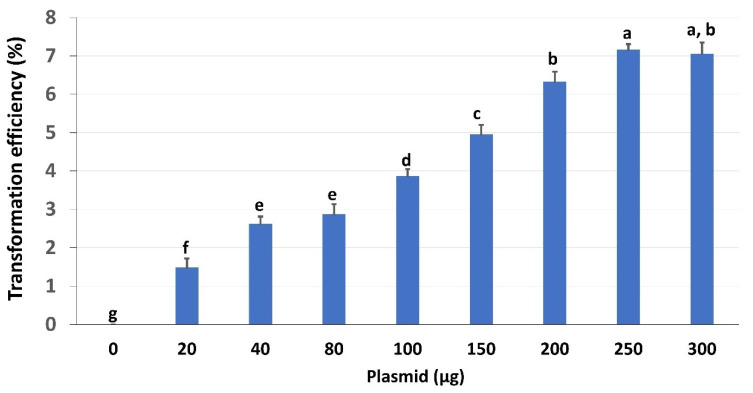
Effects of plasmid concentrations on protoplast transfection: The transformation efficiency of protoplasts cultivated with various concentrations of plasmids. The protoplast was evaluated after incubation in 50% PEG solution for 10 min. Values represent means ± SE (*n* = 7). The different letters indicate significant differences at *p* < 0.05.

The amount of plasmid concentration is also critical for protoplast TE. Using the optimized conditions (50% PEG, incubated for 5 min), we examined the effects of different concentrations of CmYLCV:GFP plasmid on TE of peanut protoplasts (Figure 4; Supplementary Figure S3). The results showed that TE increased up to 7% with the increasing amount of plasmids from 20 to 300 μg, and the concentrations between 250 μg and 300 μg plasmids yielded the highest TE (Figure 4). It is worth mentioning that the viability of protoplasts did not change due to the increase in plasmid concentration (data not shown).

### 2.5. Effects of PEG Incubation Time on Protoplast Transformation Efficiency

**Figure 5:**
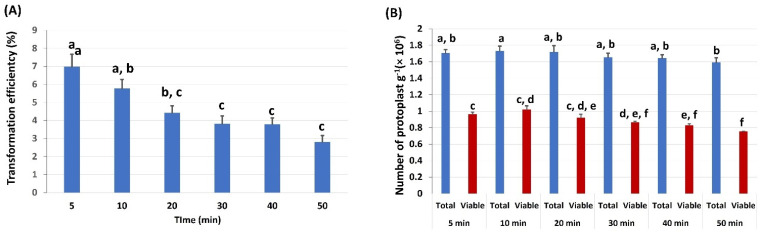
Effects of PEG incubation time on protoplast transfection: (**A**) the transformation efficiency of protoplasts cultivated with various PEG incubation times; (**B**) the effects of incubation time in PEG on protoplasts. The number of total protoplasts and viable protoplasts was counted after 24 h cultivation; 50% PEG solution was used in this experiment. Values represent means ± SE (*n* = 7). The different letters indicate significant differences at *p* < 0.05.

To identify the optimum PEG incubation time, we examined the effect of different PEG incubation times on TE and protoplast viability (Figure 5; Supplementary Figure S3). The results showed that the TE was the highest (up to 7%) after incubation for 5 min with the 50% PEG concentration (Figure 5A), and afterward (>5 min), TE decreased. The total protoplasts and viable protoplasts from these various incubation times also had a similar trend (Figure 5B). Therefore, we inferred that 5 min was the optimal PEG incubation time.

### 2.6. Selection of DNA Sequence of Ara h 2 Gene Target and Vector Construction

**Figure 6:**
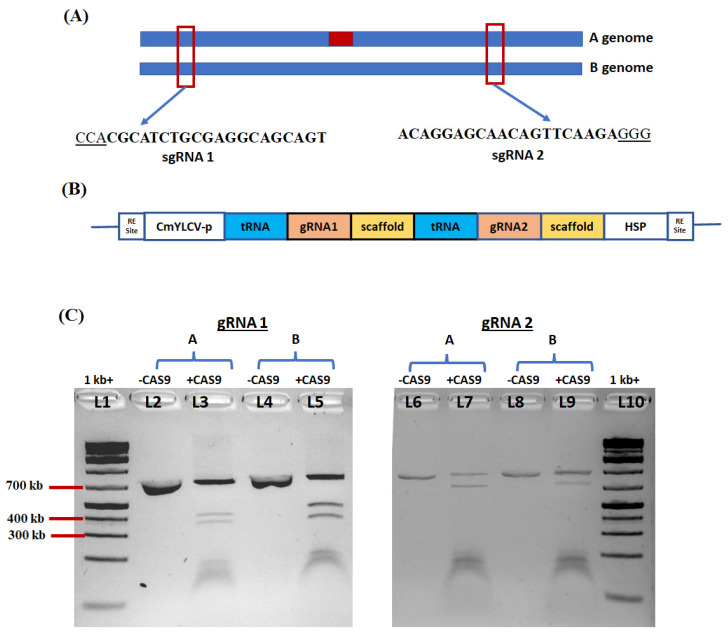
Schematic diagram of the peanut *Ara h 2* target gene copies, tRNA–sgRNAs of *Ara h 2*, and in vitro digestion of *Ara h 2* gene targets: (**A**) schematic diagram representation of peanut *Ara h 2* gene copies at A and B genome and gRNA target regions; (**B**) schematic diagram representation of tRNA–sgRNAs of *Ara h 2;* (**C**) in vitro digestion of *Ara h 2*. L1 and L10: 1kb^+^ ladders; L2: uncut *Ara h 2A* target region (genome A); L3: *Ara h 2A* target region digested with Cas9 and sgRNA1 (expected bands of 399 bp and 376 bp); L4: uncut *Ara h 2B* target region (genome B); L5: *Ara h 2B* target region digested with Cas9 and sgRNA1 (expected bands of 396 bp and 380 bp); L6: uncut *Ara h 2A* target region (genome A); L7: *Ara h 2A* target region digested with Cas9 and sgRNA2 (expected bands of 596 bp and 199 bp); L8: uncut *Ara h 2B* target region (genome B); L9: *Ara h 2B* target region digested with Cas9 and sgRNA2 (expected bands of 564 bp and 212 bp).

The coding sequence of *Ara h 2* (NM_001376217.1) was used to search for homologous sequences within the peanut reference genome database (http://peanutbase.org (accessed on 24 December 2021)), and two copies of *Ara h 2* (*Ara h 2A* and *Ara h 2B*) were identified in the A and B genomes (Figure 6A). The conserved regions for both copies were identified, amplified with allele-specific primers (Table S1), and sequenced. To increase the chance of disrupting the *Ara h 2* gene sequence, two distinct gRNAs (gRNA1 and gRNA2) were designed. The CRISPR-P program was used to identify gRNAs with the highest efficacy and the least off-target potential [32]. The polycistronic tRNA–gRNA (PTG) construct bearing the two sgRNAs was cloned into a nonbinary vector (pTrans_100). (Supplementary Figure S4 and Figure 6B). The Cas9 gene and tRNA–gRNA (PTG) were expressed under the control of the CmYLCV promoter (Supplementary Figure S4 and Figure 6B).

### 2.7. In Vitro Test of sgRNA Efficiency

In vitro ribonucleoprotein (RNP) assay for the two gRNAs targeting a PCR amplicon flanking the target site of the peanut *Ara h 2* gene was performed using the RNP complexes with purified Cas9 (Invitrogen) and synthetic gRNAs (Synthego). The negative controls had uncut PCR products, while three bands were seen for the cut amplicon with gRNA1 and gRNA2, indicating that both sgRNAs efficiently cut their target nucleotide sequences in the *Ara h 2* gene copies (Figure 6C).

### 2.8. Editing of Ara h 2 Gene in Peanut Protoplasts

To test the gene-editing efficacy of the CRISPR–Cas9 vector for *Ara h 2*, peanut protoplasts were transformed with our optimized protocol. Genomic DNA was extracted to amplify the DNA fragment containing the target site. Deep sequencing of targeted PCR products obtained from the isolated genomic DNA of each protoplast pool was used to detect the editing efficiency and patterns. The sequencing results revealed various indel mutation frequencies ranging from 0.13% to 0.8% for each CRISPR sgRNA sample (Table 1), disrupting the protein sequence (Figure S5). Notably, on plant sample S2, both sgRNAs cut both genomic copies of *Ara h 2* and deleted several nucleotides of the target genes. On the other hand, on plant sample S1, the two sgRNAs only edited genome A of the *Ara h 2* gene.

## 3. Discussion

Isolation of high yield and good quality protoplasts depends on the use of proper tissue and age of the plants [33]. For leguminous crops such as chickpea and soybean, fully expanded leaves are the best choice for protoplasts isolation [34,35]. However, our results demonstrated that the best source for protoplast isolation was unexpanded leaves from 5 days old seedlings. Furthermore, using such leaf tissues the oval-shaped cells were identified as the most successful for PEG-mediated transformation. Spherical-shaped cells were recovered from the isolation of the expanded leaves of the peanut plants; however, this type of cell failed in the PEG-mediated transformation. This may indicate that the oval-shaped cells were the true protoplasts, while the spherical-shaped cells were presumably spheroplasts [36].

Temperature is another crucial factor for maintaining the viability of the isolated protoplasts. Most plant protoplasts are stable at room temperature (23 °C–28 °C) [37]. In contrast, however, our experiments showed that all the peanut protoplasts died at 23 °C after 48 h. Therefore, we tested the viability of protoplasts at 4 °C and 13 °C. Our tests indicated that 13 °C was the ideal temperature for the PEG-mediated transformation. The optimum concentration of PEG and the duration of the PEG incubation time are other criteria that need to be considered for increasing transformation efficiency in protoplasts; it varies from plant to plant [27,37]. Our data showed that 50% of PEG and 5 min incubation time gave the best results for peanut protoplast transformation. The concentration of the plasmid is also a key factor in protoplast transformation. Different amounts of plasmids, such as 15 μg for wheat, 20 μg for rice, 30 μg sugarcane, have been reported to be the optimal amounts of DNA in their optimized protocols with TE of 70–80% for protoplast transformation [33,38,39]. Another study in oil palm protoplast achieved TE of 2.73% with 40% PEG and 50 µg plasmid, which was highest for this plant [40]. In our study with peanut protoplast, we obtained TE of 7% using 250–300 μg of CmYLCV plasmid.

Gene-editing technology has not yet been used widely in peanuts. Thus far, the only reported study of gene editing in peanuts has been the knocking out of the FAD2 gene using the CRISPR–Cas9 system through the *Agrobacterium rhizogenes*-mediated hairy root transformation [41]. However, the major limitation of the hairy root-regenerated transformants was the integration of unwanted pRi T-DNA [42]. The presence and expression of the oncogenes in pRi T-DNA may cause some problems in analyzing the phenotypic evaluations of the transgenic lines. The use of *Agrobacterium tumefaciens*-mediated transformation may overcome such problems [43]; however, the effectiveness of Cas9-gRNAs on the target gene needs to be evaluated first before generating stable transformants to increase the chance of our success. For this purpose, PEG-mediated protoplast transformation can be used.

In order to increase our chance of success, two gRNAs were designed to disrupt allergen gene function in the peanut cultivar Schubert. Due to the natural preference of the DNA repair system for non-homologous end joining (NHEJ), insertion and deletions (INDELs) are the most common type of mutations that occurred by CRISPR–Cas9 editing system [44]. We verified that all the gRNAs efficiently cut their respective allergen target site through in vitro digestion with Cas9 protein and identified two edited samples after transformation with CRISPR–Cas9 plasmid. In silico analysis revealed that all the edited plants had different amino acid changes due to deletions. For the edited sample S2, premature stop codons were generated in the coding sequence of both gene copies. Meanwhile, for the edited sample S1, the coding sequences of *Ara h 2A* completely changed due to the deletions in the two gRNA regions.

## 4. Materials and Methods

### 4.1. Plant Material

Schubert, a peanut cultivar developed by Texas A&M AgriLife Research [45], was used in this study. Schubert is a high-yielding, high-oleic acid, early maturing Spanish-type peanut cultivar with improved shellout. The peanut seedlings were grown in a greenhouse with a temperature of 32/26 °C (day/night) and a 16/8 h light–dark cycle.

### 4.2. Plasmid Preparation and Constructs

The 35S:GFP and CmYLCV:GFP vectors were used for checking the transformation efficiency in this study. Three intermediate module plasmids A, B, and C were prepared for the construction of the CRISPR–Cas9 vector of *Ara h 2* [46]. For module A, CmYLCV promoter from pMOD_A3003 (Addgene #91043) was inserted into pMOD_A0101 (Addgene #90998) in place of 35S promoter via restriction digestion and cloned using T4 Ligase (NEB, Ipswich, MA, USA) (Figure S2A,B). The pMOD_B2303 vector was used for module B. The polycistronic tRNA–gRNA (PTG) gene containing two sgRNAs sequences for *Ara h 2* [47] was synthesized and incorporated commercially into pUC57 (Genscript Biotech Ltd., Piscataway, NJ, USA). The synthesized pUC57-PTG was digested with *Pst*I and *Xho*I and cloned into the *Pst*I and *Xho*I-digested pMOD_B2303 vector (Addgene #91068) using T4 Ligase (NEB) following the manufacturer’s recommendations (Figure S2A,C). Modified pMOD_A0101, modified pMOD_B2303, and empty vector pMOD_C0000 (Addgene #91081) were assembled into a non-binary vector, pTRANS_100 (Addgene #91198) by simple Golden Gate protocol using the *Aar*I enzyme [47] (Figure S2A,D).

### 4.3. In Vitro Efficiency Test of sgRNAs

All steps were performed according to the manufacturer’s instructions for in vitro digestion of DNA with Cas9 nuclease (NEB), with a few modifications. In this case, a 27 μL reaction mixture containing 30 nM of synthesized sgRNA, 30 nM of Cas9 nuclease, and 3 μL of 10× NEB buffer 3.1 were pre-incubated for 10 min at 25 °C. Afterward, 100 ng substrate purified PCR product was added to make a total reaction volume of 30 μL and incubated at 37 °C for 1 h. After adding 1 μL of proteinase K, the reaction mixture was kept for 10 min at 56 °C, and fragment analysis was then performed using gel electrophoresis.

### 4.4. Protoplast Isolation from Peanut

Protoplasts were isolated from different tissues of 5 and 10 days old peanut seedlings according to previously published protocols [38,48], with some modifications. Briefly, tissues were cut into latitudinal strips using a sharp razor and transferred the strips into a 150-mL conical flask containing 20 mL of filter-sterilized enzyme solution (Table 2), and the flask was wrapped with aluminum foil. The strips with cell-wall-dissolving enzymes were vacuum infiltrated by applying a vacuum (~380–508 mmHg) for 30 min in the dark. Next, the strips were incubated in the dark for 5 h with gentle shaking (50 RPM) at room temperature (RT). After enzymatic digestion, 25 mL of W5 solution were added to the conical flask and then shaken gently by hand for 10 s to release the protoplasts. The protoplasts were collected into three or four 50 mL round-bottomed centrifuge tubes after filtering the mixture through 40 µm nylon meshes and washing the strips on the surface of the nylon mesh 3–5 times with W5 solution. The solution containing protoplast was centrifuged at 100× *g* for 2 min at RT in a swinging bucket rotor, and the supernatant was removed by pipetting. Protoplasts were resuspended in 10 mL of W5 solution and then collected into a 50 mL round-bottomed tube. Afterward, they were centrifuged at 100× *g* for 2 min at RT, the supernatant was removed by pipetting, and the protoplasts were then resuspended in 4 mL of MMG solution and ready for further evaluation.

### 4.5. Protoplast Counting and Viability Test

The total number of protoplasts was counted under a microscope (×100) using a hemocytometer (XB. K.25, QiuJing, Shanghai, China). For this process, 10 microliters of protoplast in MMG solution were added to the surface of the hemocytometer and carefully covered with a glass slide to avoid bubbles formation. The number of intact protoplasts in the four corners of the grid was counted under the microscope. The protoplast density was calculated as follows: protoplasts number (g^−1^) = the average count of protoplast per square ×10^4^.

Fluorescein diacetate (FDA) and propidium bromide staining (Sigma-Aldrich, St. Louis, MO, USA) were used to determine the protoplast viability according to the manufactural protocol. In this case, 1 mL each of fluorescein diacetate and propidium bromide was added to a tube containing 98 mL of water or PBS. Afterward, 10 mL of the 10× stain solution was added to 90 mL of protoplast cells and mixed well by gently tapping. After incubation for 2 min, the viability of protoplasts was determined with an Echo Revolve microscope, under ultraviolet light. The viable protoplasts were stained green, whereas the dead cells and cell debris were not stainable. The viable protoplasts ratio was calculated as follows: percentage of viable protoplasts = (green stained protoplasts determined under fluorescence microscope)/(total protoplasts observed under the bright field).

### 4.6. Protoplast Transfection

PEG-mediated transfection was performed following a previously published method [49], with some modifications. The 15 mL conical bottom tubes were coated with 5% FBS (fetal bovine serum), spun at 100× *g* for 2 min, and the FBS was removed. Next, 100 μL DNA (20–300 μg of plasmid DNA) were added to 400 µL of protoplasts suspension (2 × 10^6^ total cells), gently flicked and inverted to mix thoroughly. Afterward, 460µL of PEG-CaCl_2_ solution was added, and the tube was gently inverted several times until fully mixed and incubated at room temperature in the dark for 5–50 min. After incubation, 3 mL of W5 solution were added to stop the reaction, inverted several times gently until fully mixed, and centrifuged at 100× *g* for 2 min, and the protoplast pellet was then recovered by carefully removing the supernatant. The protoplast pellet was then resuspended with gentle inversions and minimal pipetting in 200 µL WS1 solution and incubated in the dark at room temperature. Then, protoplast viability was measured using light microscopy, and the transformation efficiency with GFP plasmid was calculated using a fluorescence microscope on a hemocytometer.

### 4.7. Deep Amplicon Sequencing

At four days post-transfection at dark condition, the peanut protoplasts were collected by centrifugation at 13000 RPM, and genomic DNA was then extracted with the CTAB protocol [50]. The Cas9–sgRNAs target sites of DNA segments were amplified with Phusion polymerase using pairs of allele-specific primers listed in Table S1. PCR was performed with an initial denaturation step of 98 °C for 30 s, followed by 32 cycles of 98 °C for 30 s, 55–58 °C for 30 s, and 72 °C for 30 s, and a final extension of 72 °C for 7 min. The PCR product was then purified by gel extraction. The site-specific primer was designed and used for the first-round amplicon PCR using the KAPA HiFi HotStart ReadyMix PCR Kit (MilliporeSigma, Burlington, MA, USA) (Table S1). PCR was performed with an initial denaturation step of 98 °C for 30 s, followed by 25 cycles of 98 °C for 30 s, 55 °C for 30 s, and 72 °C for 30 s, and a final extension of 72 °C for 5 min Next, forward and reverse barcodes for amplicon library construction were added to the PCR products for the second round of PCR with an initial denaturation step of 98 °C for 30 s, followed by 8 cycles of 98 °C for 30 s, 55 °C for 30 s, and 72 °C for 30 s, and a final extension of 72 °C for 5 min. Each sample corresponded to a unique pair of barcodes. The products of 1st and 2nd round amplicon PCR were purified using a CleanNGS kit (CleanNA, Waddinxveen, The Netherlands), according to the manufacture’s protocol. The libraries were pooled into equimolar concentrations for multiplexed sequencing on the Illumina MiSeq platform (Illumina, San Diego, CA) with 2×150 run parameters at Texas A&M Institute for Genome Sciences and Society (TIGSS) lab [51]. The obtained next-generation sequencing data were analyzed using CRIS.py [52]. Indels located around the Cas9 cleavage site (3 bp upstream of the protospacer–adjacent motif sequence) were considered to be mutations induced by Cas9.

## 5. Conclusions

An efficient gene-editing platform in peanuts needs to be established to assist in basic research in trying to understand gene functions and molecular pathways and to help speed up breeding programs in developing peanuts with improved yield, quality, and tolerance to various abiotic and biotic stresses. Our study described the success in developing an efficient protoplast isolation protocol in peanut as a testbed for optimizing genome editing using the CRISPR–Cas9 system, with the allergen gene *Ara h 2* as a test case. This strategy provides an efficient pipeline to develop gene-editing constructs for various genes or peanut transformation. Once optimized, stable transformants can be developed using *Agrobacterium*-mediated transformation or alternative delivery systems. Additionally, further optimization of the CRISPR–Cas9 system in peanuts can be explored using other editing techniques, including allele replacement, to widen the target traits and speed up the breeding progress.

## Figures and Tables

**Table 1:** Mutation analysis by targeted deep sequencing in *Ara h 2* gene.

Plant No	*Ara h 2* gRNA Target Region (5′-3′)	Type of Edit	Editing Efficiency
	*Ara h 2A* (genome A) *gRNA1* NGS results		
WT	GCTGCCCA**CGCATCTGCGAGGCAGCAGT**GGGAACTCCAA		
S1	GCTGCCCA**CGC**------**TGCGAGGCAGCAGT**GGGAACTCCAA	3 bp deletion	0.8%
S2	GCTGCCCA**CG**----------**GCGAGGCAGCAGT**GGGAACTCCAA	5 bp deletion	0.37%
	*Ara h 2B* (genome B) *gRNA1* NGS results		
WT	GCTGCCCA**CGCATCTGCGAGGCAGCAG**TGGGAACTCCAA		
S1	GCTGCCCA**CGCATCTGCGAGGCAGCAGT**GGGAACTCCAA	No edit	
S2	GCTGCCCA**CGC**--------**GCGAGGCAGCAGT**GGGAACTCCAA	4 bp deletion	0.20%
	*Ara h 2A* (genome A)*gRNA2* NGS results		
WT	GGGAGGCA**ACAGGAGCAACAGTTCAAGA**GGGAGCTCAG		
S1	GGGAGGCA**ACAGGAGCAAC-------------AGA**GGGAGCTCAG	6 bp deletion	0.14%
S2	GGGAGGCA**ACAGGAGCAACAG------AAGA**GGGAGCTCAG	3 bp deletion	0.13%
	*Ara h 2B* (genome B) *gRNA2* NGS results		
WT	GGGAGGCA**ACAGGAGCAACAGTTCAAGA**GGGAGCTCAG		
S1	GGGAGGCA**ACAGGAGCAACAGTTCAAGA**GGGAGCTCAG	No edit	
S2	GGGAGGCA**ACAGGAGCAACAG------AAGA**GGGAGCTCAG	3 bp deletion	0.16%

Nucleotides in bold fonts represent the gRNA sequence and the underlined nucleotides represent the PAM sequence.

**Table 2:** Solutions used for peanut protoplast isolation and transformation.

Solution Name	Composition
Enzyme solution	3% cellulase RS (Yakult, Tokyo, Japan), 0.1% macroenzyme, 0.5% pectinase, 0.4 M Mannitol, 20 mM KCl, and 20 mM MES (pH 5.7), 10 mM CaCl2, 0.1% BSA Special instructions: MES, mannitol, H_2_O, cellulase RS, macroenzyme, and pectinase were stirred and incubated at 55 °C for 10 min. The solution was cooled to room temperature, and CaCl_2_ and BSA were added in and gently mixed.
W5 solution	154 mM NaCl, 125 mM CaCl_2_, 5 mM KCl, and 2 mM MES (pH 5.7)
Washing and Incubation Solution (WS1)	0.5 M Mannitol, 20 mM KCl, and 4 mM MES (pH 5.7)
MMG Solution	0.4 M Mannitol, 15 mM MgCl2, and 4 mM MES (pH 5.7)
PEG–CaCl_2_ solution	0.2 M Mannitol, 0.1 M CaCl_2_, and 20–80% PEG 4000

## Data Availability

All data are available in the Supplementary Materials.

## Supplementary Materials

The following are available online at https://www.mdpi.com/article/10.3390/ijms23020837/s1.
