# Supplementary material for: Optimization of Protoplast Isolation and Transformation for a Pilot Study of Genome Editing in Peanut by Targeting the Allergen Gene Ara h 2
Source: Int J Mol Sci. 2022 Jan 13;23(2):837. doi: 10.3390/ijms23020837 (PMC8775966; doi:10.3390/ijms23020837)
Supplement: Supplementary file 1 [file ijms-23-00837-s001.zip › ijms-1550224-supplementary.pdf]

Supplementary Materials

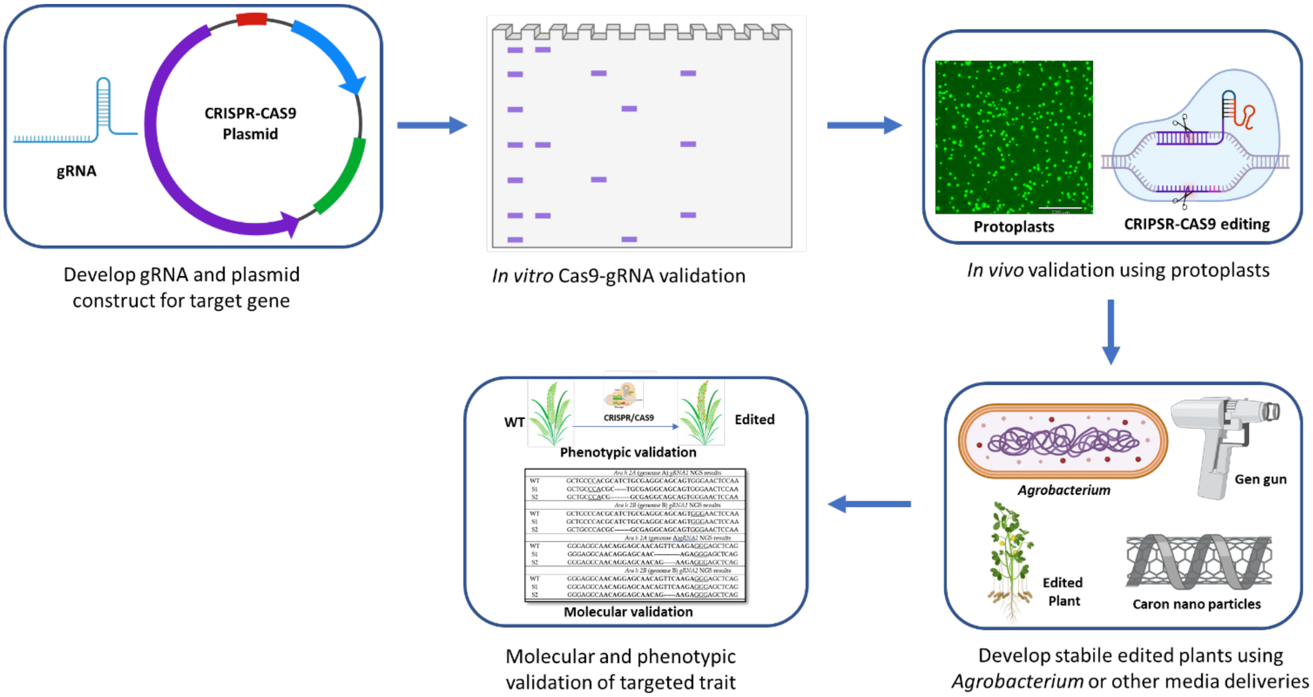

Figure S1. A Workflow for *in vitro* and *in vivo* Cas9-gRNA validation to support an efficient gene editing pipeline (diagram created by BioRender.com).

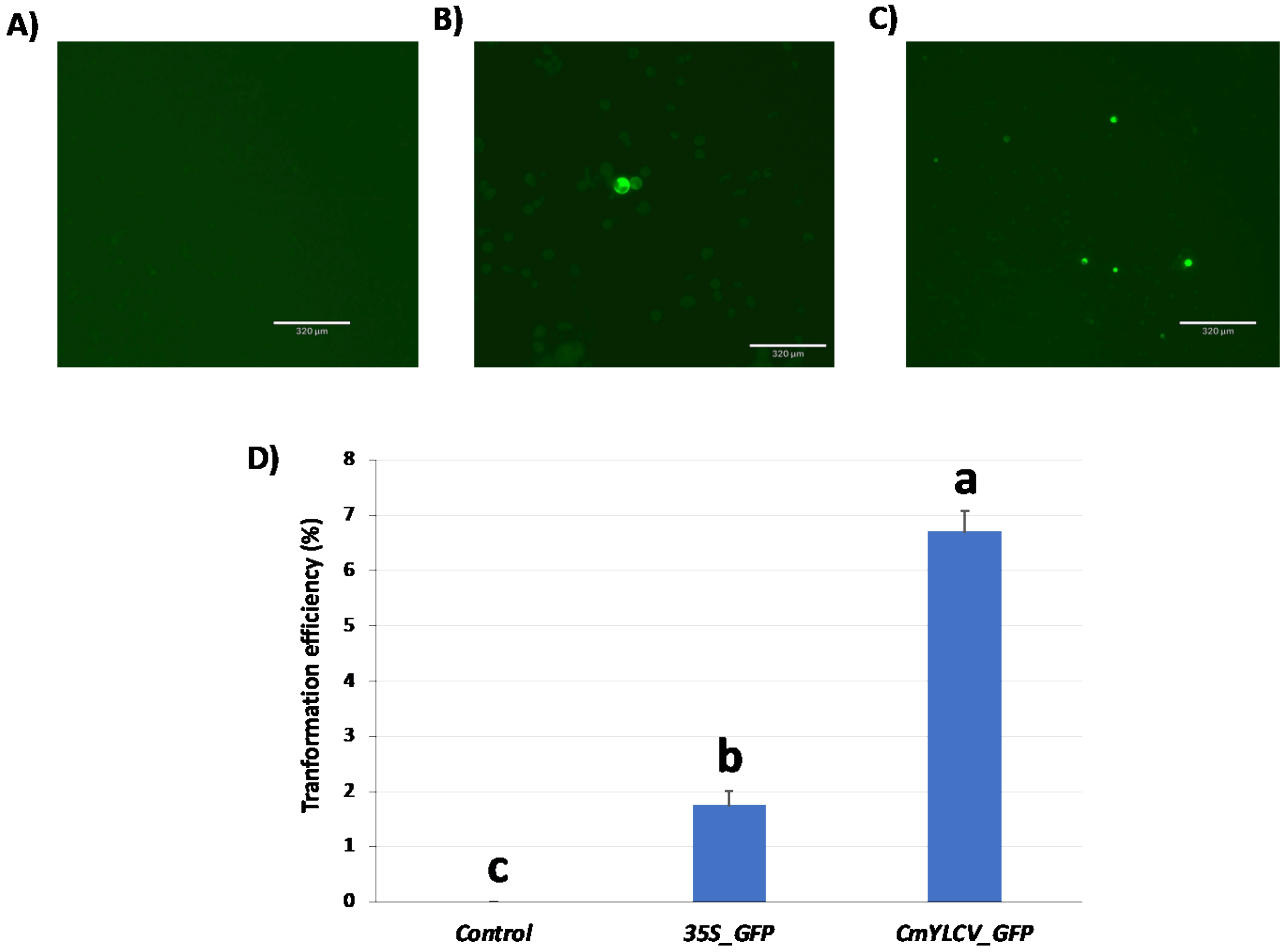

Figure S2. GFP expression under 35S and CmYLCV promoter in peanut protoplast. Micrograph of A) control protoplast (no GFP plasmid); B) protoplast with GFP expression under 35S; C) protoplast with GFP expression under CmYLCV promoter; D) The transformation efficiency (TE) of protoplasts transformed with 35S:GFP and CmYLCV:GFP plasmid. The protoplasts TE was evaluated after incubation in 50% PEG solution. Values represent means ± SE (n = 7). The different letters indicate significant differences at $P < 0.05$.

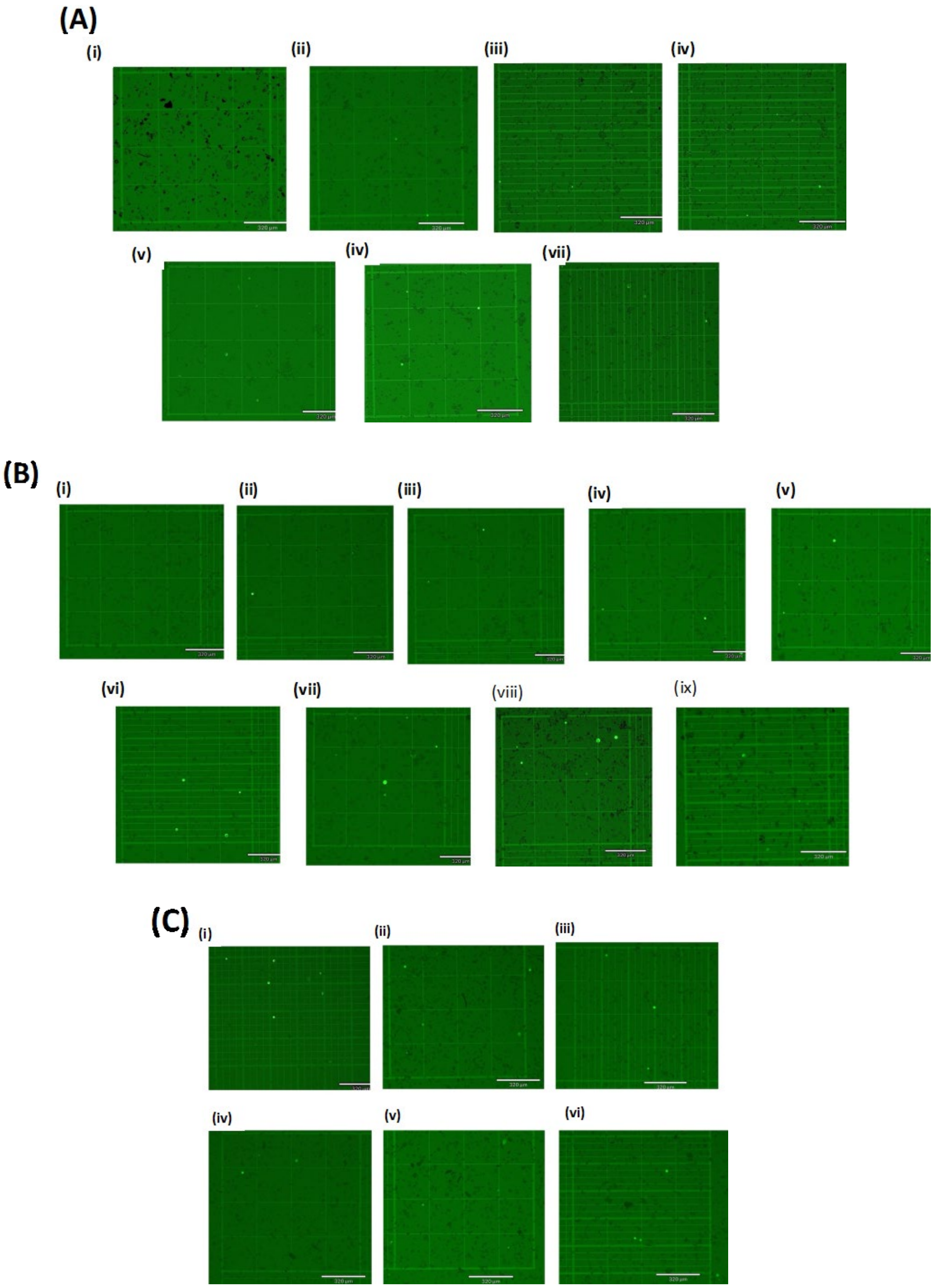

Figure S3. Effect of PEG concentration, plasmid concentrations and PEG incubation time on protoplast transfection. A) (i)-(vii) Micrographs of protoplasts expressing CmYLCV:GFP under GFP field treated with 20%, 30%, 40%, 50%, 60%, 70% and 80% PEG concentrations, respectively. The protoplasts were treated with 250 µg of CmYLCV:GFP plasmid for 5 min. B) (i)-(ix) Micrographs of protoplasts expressing CmYLCV:GFP under GFP field treated with 0 µg, 20 µg, 40 µg, 80 µg, 100 µg, 150 µg, 200 µg, 250 µg and 300 µg plasmid concentrations, respectively. In this case, the condition was 50% PEG and 5 min PEG incubation time. C) (i)-(vi) Micrographs of protoplasts expressing CmYLCV:GFP under GFP field treated with 5 min, 10 min, 20 min, 30 min, 40 min and 50 min PEG incubation time, respectively. Here, the protoplasts were incubated with 250 µg of CmYLCV:GFP plasmid and 50% PEG.

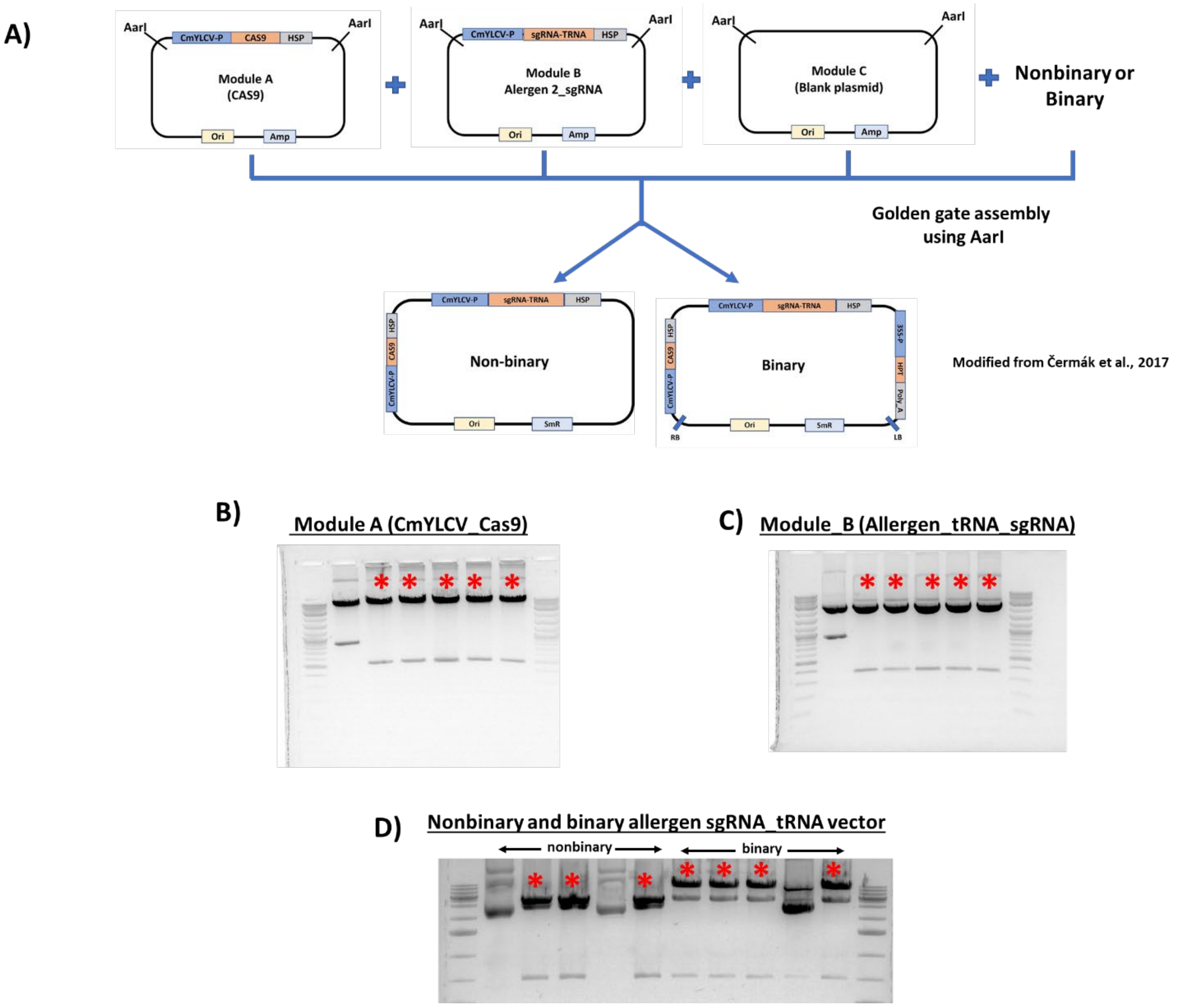

Figure S4. Cloning of Polycistronic tRNA_gRNAs (PTG) of *Ara h 2* gene into destination vector (non-binary and binary). A) Schematic diagram of golden gate assemble assembly for making CRISPR-CAS9 vector; B), C) and D) confirmation of positive clones by digestion with specific restriction enzymes. The symbol "*" indicates the positive clones of respective vectors.

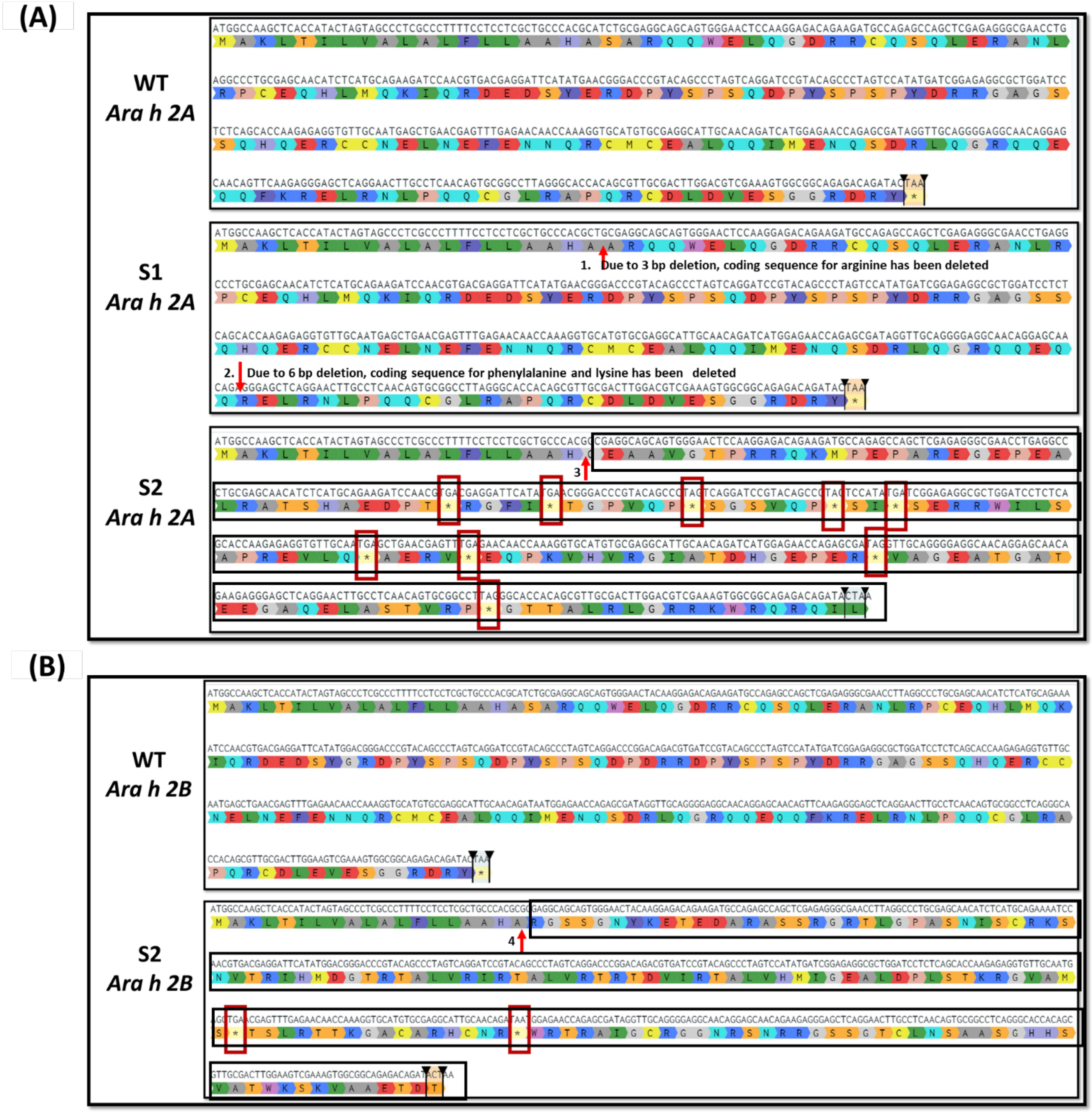

Figure S5. Changes in the coding sequence at *Arah 2* gene due to deletion in two sgRNA regions*:* A) Coding sequence of *Ara h 2A* in WT and edited sample S1 and S2. B) Coding sequence of *Ara h 2B* in WT and edited sample S1 and S2.

1↑ indicates where due to 3 bp deletion, coding sequence of arginine has been removed and 2↑ indicates where due to 6 bp deletion, coding sequence of phenylalanine and lysin has been removed in *Ara h 2A* of S2

Frameshift mutation (black block) occurred due to the mutation in the two gRNA regions for S2 samples; 3↑ and 4↑ indicates the frameshift start site in *Ara h 2A* and *Ara h 2B* of S2 edited sample. However, premature stop codons were generated in the coding sequence of both gene copies in edited sample S2. Red block indicates the premature stop codon position in the coding region of *Ara h 2A* and *Ara h 2B* in S2.

Table S1. Primers used in this study.

Primer	Sequence (5′-3′)
Arah 2AF	GAAGGTGCATTAAACATTGAACATGTG
Arah 2AR	ATGATCTTTATTATTACCAAAACTAACATAA
Arah 2BF	GAAGGTGCATTAAACATTGAACATCTC
Arah 2BR	ATGATCTTTATTATTACCAAAACTAACATTA
Arah2A_NGS_1F	TCGTCGGCAGCGTCAGATGTGTATAAGAGACAGCACCACACACTCTTCAATACACATTC
Arah2A_NGS_1R	GTCTCGTGGGCTCGGAGATGTGTATAAGAGACAGTCCTGACTAGGGCTGTACGGG
Arah2A_NGS_2F	TCGTCGGCAGCGTCAGATGTGTATAAGAGACAGGATCCTCTCAGCACCAAGAGAGGT
Arah2A_NGS_2R	GTCTCGTGGGCTCGGAGATGTGTATAAGAGACAGCAAGTCGCAACGCTGTGGTG
Arah2B_NGS_1F	TCGTCGGCAGCGTCAGATGTGTATAAGAGACAGTACCTCACATGCAAAATCCCTC
Arah2B_NGS_1R	GTCTCGTGGGCTCGGAGATGTGTATAAGAGACAGGCCTAAGGTTCGCCCTCTCG
Arah2B_NGS_2F	TCGTCGGCAGCGTCAGATGTGTATAAGAGACAGAAGGTGCATGTGCGAGGCAT
Arah2B_NGS_2R	GTCTCGTGGGCTCGGAGATGTGTATAAGAGACAGTAGCTTATATATAAGCTATTTTCTTT
